# N6-methyladenosine modification of mRNAs in retinal ischemia-reperfusion in mice

**DOI:** 10.1080/15592294.2026.2700836

**Published:** 2026-07-12

**Authors:** Jingying Liang, Yuke Li, Yuwen Wen, Zhidong Li, Caibin Deng, Yehong Zhuo, Yangyang Li, Yingting Zhu

**Affiliations:** State Key Laboratory of Ophthalmology, Zhongshan Ophthalmic Center, Sun Yat-sen University, Guangdong Provincial Key Laboratory of Ophthalmology and Visual Science, Guangzhou, China

**Keywords:** Methylation, mRNA, retinal ischemia-reperfusion, retina

## Abstract

Retinal ischemia-reperfusion injury (RIR) is the main pathogenic mechanisms of acute glaucoma, diabetic retinopathy, central retinal vein occlusion. As a common post-transcriptional modification of eukaryotic RNAs, N6-methyladenosine (m^6^A) is associated with the pathogenesis of different diseases, including angiogenesis, through the regulation of RNA metabolism and functions. The aim of this study was to identify the potential relevance of m^6^A RNA methylation in pathogenesis of RIR. A total of 10,851 mRNAs and 23,270 associated m^6^A methylation modified peaks were identified in the RIR group. Similarly, 10,391 mRNAs and 22,935 associated m^6^A methylation modified peaks were detected in the Sham group. MeRIP-seq identified 3,871 RIR-specific m^6^A peaks and 3,624 Sham-specific m^6^A peaks, in addition to 19,399 shared peaks between groups. Gene ontology (GO) analysis showed that hypermethylated mRNAs were enriched in cellular process, cellular anatomical entity, and binding, while hypomethylated mRNAs were enriched in synaptic signaling, synapse, and gated channel activity. Kyoto Encyclopedia of Genes and Genomes (KEGG) pathway analysis indicated that hypermethylated mRNAs were involved in tight junction, hippo signaling pathway, and PI3K-Akt signaling pathway, while hypomethylated mRNAs were involved in Neuroactive ligand-receptor interaction, glutamatergic synapses, cholinergic synapses. Joint analysis identified mRNAs with differential m^6^A methylation and expression simultaneously. Among them, the expression patterns of *Irx4*, *Kdr*, and *Lyz2* were confirmed by RT-qPCR to be consistent with the sequencing results. The results revealed an altered m^6^A epitranscriptome in RIR retinas. These methylated RNAs may act as novel modulators and targets in RIR.

## Introduction

RIR is a prevalent pathological event triggered by transient blood flow occlusion and subsequent reperfusion. This pathogenesis is associated with many ophthalmic diseases, such as acute glaucoma [1], diabetic retinopathy [2] and central retinal artery occlusion [3], which are vital causes of blindness worldwide. However, previous clinical trials (phase II/III) have shown that neuroprotective drugs failed to achieve significant inhibition of neural impairment [4], further clarification of the pathogenesis of RIR is necessary to facilitate the development of novel therapeutic targets and safer, more effective treatments.

Recent studies have revealed that, beyond classical genetic and protein-level regulation, epigenetic modifications – particularly at the RNA level – play critical roles in ischemic and neurodegenerative diseases. RNA epigenetic modification represents a highly conserved post-transcriptional regulatory mechanism that modulates diverse genetic processes in eukaryotes. As the most common and conserved epigenetic modification in mRNA in most eukaryotic species [5,6], m^6^A modification is dynamically regulated by m^6^A methylases, demethylases and reader proteins [7] without changing the based sequence. Through these post-transcriptional effects, m^6^A modification profoundly influences gene expression programs and cellular homeostasis [8,9], thereby participating in the development of various pathological processes, including aging, neurological diseases [10], renal and cerebral ischemia-reperfusion injury [11,12]. Increasing evidence has also linked aberrant RNA methylation to ophthalmic diseases such as pseudoexfoliation glaucoma [13,14], diabetic retinopathy [15] and keratitis [16], highlighting the potential involvement of m^6^A in ocular pathological process. The role of m^6^A modification in the pathogenesis of RIR remains uncertain. Therefore, this study sought to characterize the global m^6^A methylation profiles of mRNAs in retinas from the RIR mouse model to uncover potential regulatory mechanisms and underlying this process.

## Methods

### Animals

Female C57BL/6J mice (6–8 weeks old) were obtained and housed under standardized laboratory conditions at the Animal Center of Zhongshan Ophthalmic Center, Sun Yat-sen University. All animal experiments were approved by the Animal Care and Use Committee of the State Key Laboratory of Ophthalmology, Zhongshan Ophthalmic Center, Sun Yat-sen University (Approval No. Z2025050; approved on 13 June 2025). All procedures were conducted in accordance with the institutional guidelines for the humane treatment of laboratory animals, the Principles of Laboratory Animal Care, and the ARVO Statement for the Use of Animals in Ophthalmic and Vision Research. Animals were monitored regularly throughout the study for signs of distress or adverse events.

### Establishment of animal model

Anesthesia was induced by intraperitoneal injection of pentobarbital (60 mg/kg). For surface anesthesia, 0.5% tetracaine hydrochloride was applied topically to the corneas, and 1% tropicamide was to achieve mydriasis. Animals were randomly assigned to each group. Continuous irrigation of the anterior chamber with sterile normal saline was performed using a 23-gauge needle to maintain the intraocular pressure (IOP) at 100 mmHg for 60 minutes. IOP was monitored using the tonometer (Icare TONOLAB tonometer TV02) to ensure consistency. The contralateral eye without IOP elevation functioned as the Sham. After removal of the needle, retinal blood flow was restored. Tobramycin ointment was applied to both sides of the eye to prevent bacterial infection. After the procedure, the mice were placed on a temperature-controlled heating pad to maintain their body temperature. Once fully recovered from anesthesia, the mice were returned to their cages and housed in an animal facility with controlled temperature and humidity. Mice were euthanized at 1, 3, and 7 days after the procedure by isoflurane overdose, in accordance with institutional animal care guidelines and the American Veterinary Medical Association (AVMA) Guidelines for the Euthanasia of Animals. The retina was carefully dissected from the eyecup under a stereomicroscope, and the underlying choroid tissues were removed as thoroughly as possible.

### RNA extraction

The Universal RNA Purification Kit (EZBioscience, China) was used to total RNA extraction from retinal tissue samples 3 days after RIR according to standard protocols. RNA concentration and purity were evaluated by the NanoDrop ND1000 spectrophotometer (Thermo Scientific).

### Methylated RNA immunoprecipitation sequencing (MeRIP-seq)

Retinal tissues from multiple eyes were pooled to generate sufficient RNA for sequencing. The m^6^A-IP-seq service was performed by CloudSeq Inc. (Shanghai, China), which was not involved in animal modelling or experimental design. Total RNA was immunoprecipitated using the GenSeq® m^6^A-IP Kit (GenSeq Inc.) according to the manufacturer’s instructions. Briefly, the RNA was randomly fragmented to approximately 200 nt using RNA Fragmentation Reagents. Protein A/G magnetic beads were incubated with anti-m^6^A antibody at room temperature for 1 hour to allow antibody conjugation. The fragmented RNA was then incubated with the antibody-conjugated beads at 4°C for 4 hours with gentle rotation. After incubation, the RNA – antibody complexes were thoroughly washed, and the bound RNA was eluted and purified. RNA libraries from both the immunoprecipitated (IP) and input samples were prepared using the GenSeq® Low Input Whole RNA Library Prep Kit (GenSeq Inc.) following the manufacturer’s instructions. Library quality was evaluated using an Agilent 2100 Bioanalyzer (Agilent Technologies), followed by high-throughput sequencing.

### MeRIP-seq bioinformatic analysis

Raw reads (Raw Data) are generated aftersequencing on a sequencer, image analysis, base identification and QC. Q30 was first used for quality control. Then, cutadapt software (v1.9.3) was used to remove splice information and remove low quality reads to obtain high quality clean reads. The clean reads were matched to the reference genome using Hisat2 software (v2.0.4). Methylated genes in each sample were then identified using MACS software (1.4.2). For peak overlap analysis, peaks identified in individual samples within the same group were merged to generate group-level peak sets, and the overlap between groups was visualized using Venn diagrams. Differential methylation gene identification was performed using diffReps software (1.55.6). Peaks with |fold change (FC)| ≥2 and false discovery rate (FDR) <0.05 were considered significantly differentially methylated. The peaks located on exons were screened using our own programs and annotated accordingly. GO and Pathway analyses were performed for differentially methylated mRNA.

### RNA sequencing

RNA sequencing service was provided by CloudSeq Inc. (Shanghai, China). Briefly, total RNA was extracted with Trizol (Invitrogen, Carlsbad, CA, USA), and subjected to ribosomal RNA (rRNA) removal using GenSeq® rRNA Removal Kit (GenSeq, Inc., Shanghai, China). Then, the rRNA- depleted samples were used for library construction with GenSeq® Directional RNA Library Prep Kit (GenSeq, Inc., Shanghai, China) by following manufacturer’s recommendations. The RNA was fragmented into ~300 nt in length. The first-strand cDNA was synthesized from the RNA fragments by reverse transcriptase and random hexamer primers, and the second-strand cDNA was synthesized in 2nd Strand Synthesis Buffer with dUTP Mix. Then, the double stranded cDNA fragments were subjected to end-repair and dA-tailing, followed by adapter ligation. The adapter-ligated DNA samples were PCR amplified and purified to obtain sequencing libraries. Finally, the libraries were sequenced with sequencer on the paired-end 150 bp mode.

### RNA-seq bioinformatic analysis

Raw reads (Raw Data) are generated after sequencing on a sequencer, image analysis, base identification and QC. Q30 was first used for quality control. Then, cutadapt software (v1.9.3) was used to remove splice information and remove low quality reads to obtain high quality clean reads. High quality reads were compared to the reference genome using HISAT2(v2.0.4). And then HTSeq (v0.9.1) was used to obtain the original count number at gene level as mRNA expression profile. edgeR (v3.16.5) was used to standardize and calculate the multiple change and *p*-value between the two groups of samples, and screen differentially expressed mRNA. Different mRNA associated genes were analyzed by GO and KEGG. Differentially methylated transcripts identified by MeRIP-seq were integrated with differentially expressed genes identified by RNA-seq to perform the combined methylation-expression analysis.

### Bioinformatic analysis of differentially methylated genes (DMGs)

Gene Ontology (GO) enrichment analysis of differentially methylated genes was conducted using the topGO package. Pathway enrichment analysis was performed based on the latest KEGG database (www.genome.jp/kegg). Statistical significance of enriched KEGG pathways was determined using Fisher’s exact test.

### Reverse transcription quantitative polymerase chain reaction (RT-qPCR)

Total RNA was reverse-transcribed into complementary DNA (cDNA) using the PrimeScript RT Master Mix reagent kit (Accurate Biology, China), according to the manufacturer’s instructions. All cDNA samples were frozen at −20°C until used. SYBR Premix Ex Tag (Accurate Biology, China) was used for RT-qPCR. Glyceraldehyde-3-phosphate dehydrogenase (GAPDH) was used as the endogenous reference gene for normalization of target gene expression. All qPCR reactions were performed using a LightCycler 480 Real-Time PCR System (Roche, Basel, Switzerland). Three independent biological replicates were analyzed for each group, and each biological sample was measured in technical triplicate. The primer sequences are provided in Table 1.

**Table 1.** Primer sequences for RT-qPCR.

Gene	Forward Primer (5 ´→3 ´)	Reverse Primer (5 ´→3 ´)
*Gapdh*	GGAGAGTGTTTCCTCGTCCC	ATGAAGGGGTCGTTGATGGC
*Alkbh5*	ATTGCCACCCAGCTATGCTT	AGACCGCCGGTTTTCTTCTT
*Fto*	GTGTTTTGGCTGGCTCACAG	GTCGCCATCGTCTGAGTCAT
*Mettl3*	CGTAGTGATAGTCCCGTGCC	ATCAGTGGGCAAGGTCAAGG
*Mettl5*	AGCTGCTGAATGGAAAGTCAAG	TTAGGTCCACTTCGATGTCCACAG
*Ythdc2*	TGACCAGTACGGAAAGAGCC	GGTCATCATTGCATGAGCTGT
*Irx4*	CATCTGGTCCTTGGCACACA	GGAGGGAAACTCAGTCTGGC
*Lyz2*	ACAACCGTGGAGACCAAAGC	TTGATCCCACAGGCATTCACA
*Kdr*	TGGGCAGTCAAGTCCGAATC	GTTGGTGAGGATGACCGTGT

### m^6^A dot blot analysis

Total RNA was isolated from retinal tissues and denatured at 95°C for 3 min. Dilutions of RNA (0.5 μg) were spotted onto nitrocellulose membranes. Membranes were blocked with 1% BSA in PBST for 1 h and incubated with an anti-m^6^A antibody(1:1000; YA3446, MedChemExpress, USA) 1.5 h at room temperature according to the manufacturer’s protocol. After incubation with HRP-conjugated secondary antibodies(1:5000; HA-P8001, MedChemExpress, USA), immunoreactive signals were detected using Enhanced Chemiluminescent reagents(NCM Biotech, P10060). A duplicate membrane stained with 0.02% methylene blue served as a loading control for RNA normalization.

## Results

### Alterations of m^6^A-related enzymes on day 1,3,7

**Figure 1.**
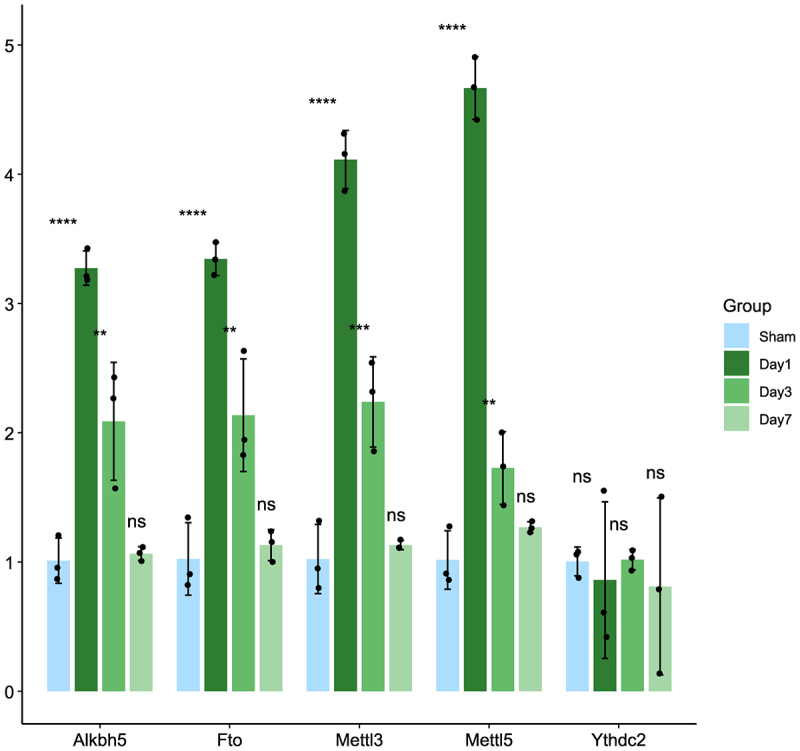
Differential expression of m^6^A regulatory genes in RIR. Relative mRNA expression levels of key m^6^A regulatory genes, Alkbh5, Fto, Mettl3, Mettl5, and Ythdc2, were determined by RT-qPCR at different time points (Day 1, Day 3, Day 7) following RIR injury, compared to the Sham group (*n* = 3). Data are presented as mean ± SEM. *p < 0.05, **p < 0.01, ***p < 0.001, ****p < 0.0001; ns, not significant.

RT-qPCR was used to test the expression changes of m^6^A methyltransferase (*Mettl5*, *Mettl3*), demethylases (*Alkbh5*, *Fto*) and binding protein (*Ythdc2*) at different time points post-intervention (Figure 1). *Mettl5* and *Mettl3* exhibited a similar expression pattern, peaking on Day 1 with highly significant upregulation (*p* < 0.0001). On Day 3, their expression levels showed a significant decrease but remained elevated above baseline (*p* = 0.0290 and *p* = 0.0100), before also returning to baseline levels by Day 7. *Alkbh5* and *Fto* both showed significant upregulation on Day 1 compared to the Sham group (*p* = 0.0001 and *p* = 0.0010, respectively), with their expression levels decreasing significantly on Day 3 (*p* = 0.0410 and *p* = 0.0270). By Day 7, the expression of both genes had returned to baseline levels with no significant difference from the Sham group. However, *Ythdc2* showed no significant change between Sham and RIR group at each day. These findings collectively suggest that the expression of these four enzymes is acutely and transiently upregulated following RIR injury, with the peak response occurring on Day 1 and gradually subsiding by Day 7.

Collectively, these results showed that multiple m^6^A regulators were markedly upregulated at the immediate stage of RIR injury (Day 1). During the acute phase (Day 3), sustained alterations in several m^6^A regulators were observed. Subsequently, the expression levels of most m^6^A regulators gradually returned toward baseline by Day 7.

### Overview of m^6^A RNA methylation in mouse retina after RIR

**Figure 2.**
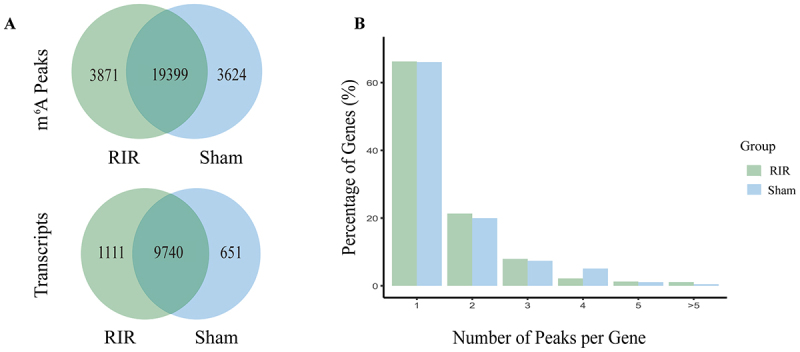
Overview of m^6^A methylation modification in the retinas of RIR and Sham. (A) Venn diagram illustrating the overlap of m^6^A peaks in mRNAs between the two groups. (B) Proportion of genes harboring different numbers of m^6^A peaks in the two groups. The majority of genes harbor only one m^6^A peak.

MeRIP-seq analysis of retinas revealed 3,871 distinct m^6^A peaks across 1,111 mRNA transcripts in RIR group and 3,624 distinct m^6^A peaks across 651 mRNA transcripts in Sham group. In addition, 19,399 shared m^6^A peaks within 9,740 mRNA transcripts were identified between the two groups (Figure 2(A)). The distribution of m^6^A peaks across transcripts was further analyzed, revealing that approximately 60% of methylated mRNAs in both groups carried a single m^6^A peak (Figure 2(B)).

**Figure 3.**
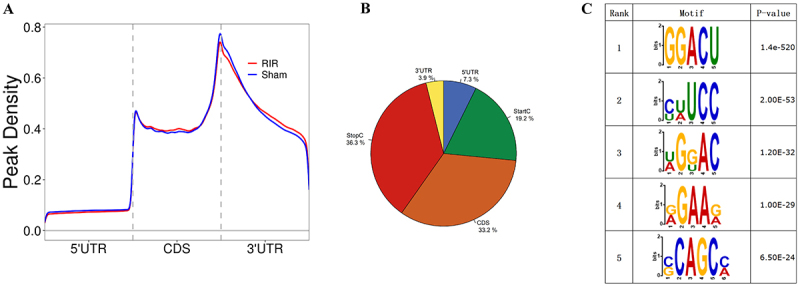
Overview of m^6^A methylation within mRNAs in RIR and Sham. (A) Metagene plots showing the enrichment of m^6^A peaks along transcripts in two groups. (B) Pie charts showing the percentage of m^6^A peaks in five nonoverlapping segments of mRNA transcripts of RIR group. (C) Motif analysis of all differentially methylated m^6^A sites of mRNAs between the RIR and Sham groups revealed enrichment of the RRACH consensus sequence.

Metagene plot revealed the identified m^6^A peaks were mainly enriched in the coding sequence (CDS), especially proximal to the stop codon (StopC), consistent with previous reports17. Notably, the RIR group exhibited a different peak density within the CDS and 3’untranslated regions (3’UTR) regions compared to the Sham group, suggesting enhanced m^6^A modification in these transcript regions under RIR-related conditions (Figure 3(A)). This may imply altered post-transcriptional regulation mediated by m^6^A in RIR pathogenesis. The distribution of m^6^A peaks across different transcript segments is shown in Figure 3(B). In RIR group, the major part of m^6^A peak were located in StopC regions (36.3%), and CDS regions (33.2%), indicating a predominant enrichment of m^6^A modifications in StopCs and CDSs. The start codon region (StartC) accounted for 19.2% of the peaks, followed by the 5’UTR (7.3%), and the 3’UTR (3.9%). These results suggest that while m^6^A sites are distributed throughout the transcripts, the StopCs and CDSs remain major hotspots for m^6^A deposition.

To identify the canonical m^6^A RRACH consensus motif (*R* = G or A; H = A, C, or U), an unbiased search for enriched motifs in regions surrounding the m^6^A peaks was performed, revealing significant enrichment of the GGAC sequence (Figure 3(C)).

### Chromosomal and transcript-level patterns of m^6^A Dysregulation in RIR

**Figure 4.**
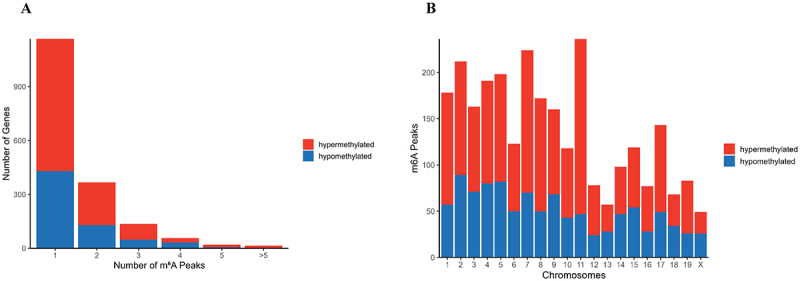
Histograms showing the distribution characteristics of m^6^A peaks. (A) Counts of m^6^A peaks per transcript in RIR. (B) Chromosomal distribution of differentially methylated m^6^A peaks among the three chromosomes showing the greatest numbers of hypermethylated and hypomethylated peaks.

**Figure 5.**
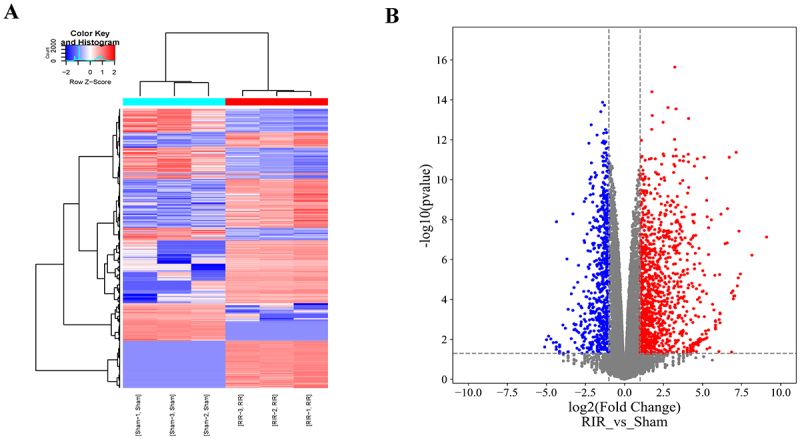
Integrative analysis of mRNA peaks with differential methylation. (A) Hierarchical clustering analysis of differentially expressed mRNAs between the RIR and Sham groups. (B) Volcano plot showing hyper- and hypomethylated mRNAs in RIR retinas compared to Sham retinas.

A total of 1762 mRNAs (1111 hypermethylated and 651 hypomethylated) were differentially m^6^A-methylated with statistical significance in the retinas of the RIR and Sham groups (|FC| ≥2 and *p* < 0.05). A predominant fraction of the altered methylated mRNAs (63.08%) were significantly hypermethylated. Furthermore, differentially hypomethylated m^6^A peaks were detected on nearly all chromosomes, with the exception of ChrY. The distribution analysis showed that chromosomes 2, 7, and 11 harbored the largest proportions of hypermethylated and hypomethylated m^6^A peaks across mRNA transcripts (Figure 4(B)). Moreover, hierarchical clustering analysis demonstrated the relationships between Sham and RIR groups, which were grouped according to similarities in their m^6^A methylation profiles (Figure 5(A)). The m^6^A sites exhibiting the largest fold changes, including both hyper- and hypomethylated sites in mRNAs, are listed in Table 2.

**Table 2.** Differentially expressed m^6^A-modified mRNAs.

			Methylation				Expression						
chrom	transcript_id	GeneName	Foldchange	*P*_value	FDR	Regulation	logFC	logCPM	F	*p*Value	FDR	Regulation	catalog
chr5	ENSMUST00000113516	*Kdr*	7455.3	2.30309E-08	2.17946E-06	down	1.0132589	8.6480942	111.16452	8.44E-06	8.72E-05	up	startC
chr13	ENSMUST00000022095	*Irx4*	5693.7	2.18224E-10	5.01985E-08	down	−2.537423	0.5919034	24.036606	0.0005162	0.002897	down	stopC
chr2	ENSMUST00000099992	*Pde11a*	4235.6	8.0259E-09	9.06136E-07	down	−1.963879	2.0568858	71.659532	2.94E-07	5.47E-06	down	startC
chr7	ENSMUST00000185406	*Muc2*	4102.6	1.91233E-09	2.81848E-07	down	−1.585739	2.4927001	44.194657	1.88E-05	0.0001718	down	CDS
chr9	ENSMUST00000034869	*Isl2*	3478.1	6.89296E-09	8.01138E-07	down	−2.228098	0.2154189	27.016905	9.31E-05	0.0006793	down	stopC
chr3	ENSMUST00000054599	*Sprr1a*	3063.3	4.24634E-08	3.66537E-06	up	7.3286209	0.8625045	95.278173	3.82E-08	1.08E-06	up	stopC
chr10	ENSMUST00000020161	*Arg1*	2728.3	2.82119E-08	2.58029E-06	up	7.2319687	2.9219633	100.8744	2.10E-05	0.0001882	up	stopC
chr10	ENSMUST00000092163	*Lyz2*	124.40752	8.9934E-09	9.9206E-07	up	5.5669332	6.1258389	987.52773	1.03E-08	3.95E-07	up	CDS
chr10	ENSMUST00000092163	*Lyz2*	73.820738	2.71876E-08	2.49797E-06	up	5.5669332	6.1258389	987.52773	1.03E-08	3.95E-07	up	startC
chr17	ENSMUST00000044804	*Cdsn*	6874.5	5.27622E-10	9.92653E-08	up	2.1345785	0.7279211	36.986727	1.70E-05	0.000158	up	stopC
chr17	ENSMUST00000044804	*Cdsn*	100.6	1.36444E-07	1.00408E-05	up	2.1345785	0.7279211	36.986727	1.70E-05	0.000158	up	3UTR
chr7	ENSMUST00000106267	*Stx1b*	3433.3	1.94619E-11	7.4305E-09	up	−1.172184	6.8369741	360.07153	2.77E-12	1.88E-09	down	3UTR
chr7	ENSMUST00000106267	*Stx1b*	2282.6	5.03408E-12	2.55825E-09	up	−1.172184	6.8369741	360.07153	2.77E-12	1.88E-09	down	stopC
chr7	ENSMUST00000106267	*Stx1b*	1912	2.16879E-12	1.47066E-09	up	−1.172184	6.8369741	360.07153	2.77E-12	1.88E-09	down	3UTR
chr7	ENSMUST00000106267	*Stx1b*	1171.8	1.3198E-10	3.34582E-08	up	−1.172184	6.8369741	360.07153	2.77E-12	1.88E-09	down	3UTR
chr7	ENSMUST00000120537	*Bcl3*	853.26829	1.23403E-08	1.29165E-06	down	2.2039714	4.8909975	266.52736	9.49E-09	3.70E-07	up	stopC
chr3	ENSMUST00000200585	*Fgf2*	827.68224	9.02831E-10	1.53053E-07	down	1.4854721	8.0836241	151.29946	1.60E-05	0.0001504	up	3UTR
chr14	ENSMUST00000130697	*Irf9*	460.9	2.22053E-14	3.88116E-10	down	1.0396393	6.1603893	42.180702	0.0009065	0.0046348	up	CDS
chr14	ENSMUST00000130697	*Irf9*	393.08108	5.59103E-10	1.03775E-07	down	1.0396393	6.1603893	42.180702	0.0009065	0.0046348	up	3UTR

### Biological functions and associated pathways of significantly altered m^6^A-modified genes

**Figure 6.**
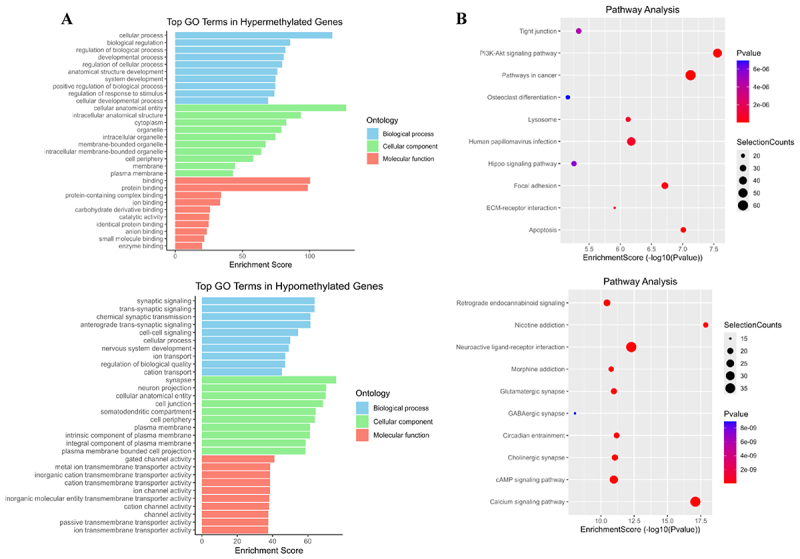
Histograms showing the significantly enriched GO terms of genes exhibiting concurrent m^6^A modification and expression changes in RIR. (A) Significantly enriched GO terms for the hypermethylated genes and hypomethylated genes. (B) Significantly enriched KEGG terms for the hypermethylated genes and hypomethylated genes.

GO analysis was performed to predict the biological functions of significantly differentially m^6^A-modified mRNAs (|FC| ≥2 and FDR < 0.05). The most significantly enriched GO terms among hypermethylated transcripts included cellular process (ontology: biological process), cellular anatomical entity (ontology: cellular component), binding (ontology: molecular function) (Figure 6(A)). The GO terms most enriched with hypomethylated mRNAs included regulation of neurotransmitter receptor diffusion trapping, cholinergic synapse, and voltage-gated calcium channel activity involved in regulation of presynaptic cytosolic calcium levels (Figure 6(A)).

KEGG pathway analysis was performed to identify the signaling pathways associated with significantly differentially m^6^A-modified mRNAs (|FC| ≥2 and FDR < 0.05). Hypermethylated transcripts were predominantly enriched in pathways including ECM-receptor interaction and tight junction (Figure 6(B)), and the pathways most enriched with hypomethylated mRNAs were Nicotine addiction, Calcium signaling pathway, Neuroactive ligand-receptor interaction (Figure 6(B)). These results suggest that hypermethylated mRNAs are predominantly involved in cellular structural organization and extracellular matrix – related processes, whereas hypomethylated mRNAs are mainly associated with synaptic transmission and neuronal communication.

### mRNA expression profiles associated with m^6^A methylation

**Figure 7.**
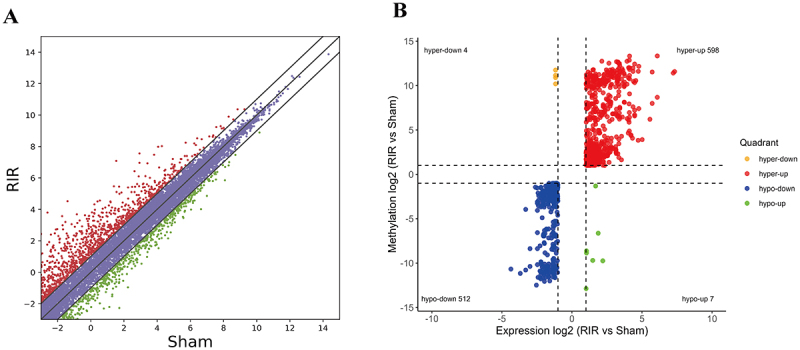
Integrated analysis of the m^6^A methylome and RNA transcriptome between RIR and Sham group. (A) Scatter plots and hierarchical clustering of differentially expressed genes between RIR group and Sham group (|FC| ≥2 and *p* < 0.05). (B) Four-quadrant plots depicting the association between m^6^A methylation and mRNA expression across the two groups.

Transcriptomic profiles of altered genes in the retinas of RIR group were analyzed by RNA-seq (Figure 7(A)). Combined analysis of the data from MeRIP-seq and RNA-Seq identified genes that showed concurrent significantly in both m^6^A modification and mRNA abundance in RIR group compared with Sham group. Four types of interaction were found (Figure 7(B)): 1. upregulated and hypermethylated mRNAs (598 peaks, including *Lyz2*); 2. downregulated and hypermethylated mRNAs (4 peaks, including *Stx1b*); 3. upregulated and hypomethylated mRNAs (7 peaks, including *Kdr*); 4. downregulated and hypomethylated mRNAs (512 peaks, including *Irx4*).

### Validation of differentially expressed and mRNA by RT-qPCR & m^6^A dot blot

**Figure 8.**
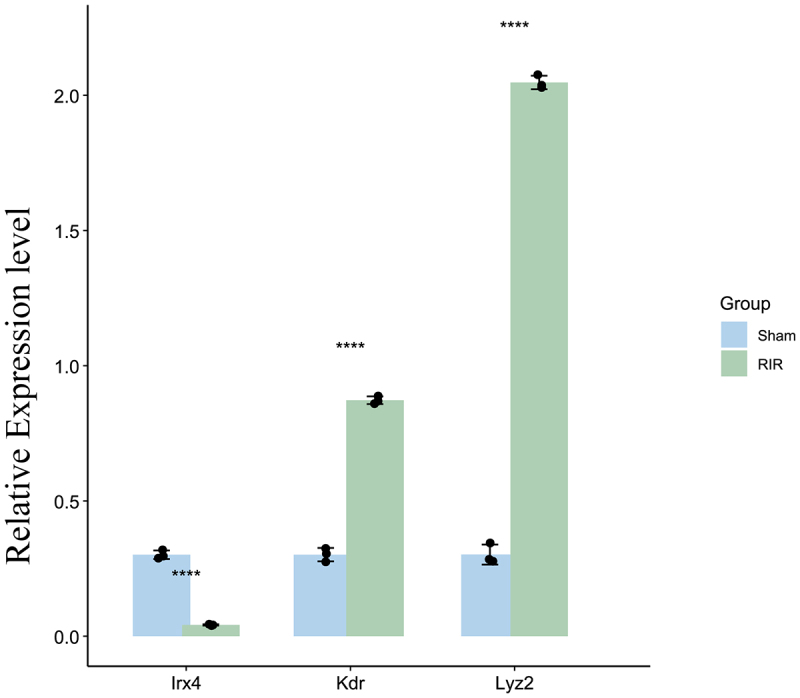
RT-qPCR validation of differentially expressed transcripts. Irx4, Kdr, Lyz2 were examined in mice with RIR (*n* = 3) and Sham (*n* = 3) by RT-qPCR and normalized to Gapdh. The student’s t-test was used to assess the differences in each gene between RIR group and Sham group. Data are presented as mean ± SEM. *p < 0.05, **p < 0.01, ***p < 0.001, ****p < 0.0001; ns, not significant.

**Figure 9.**
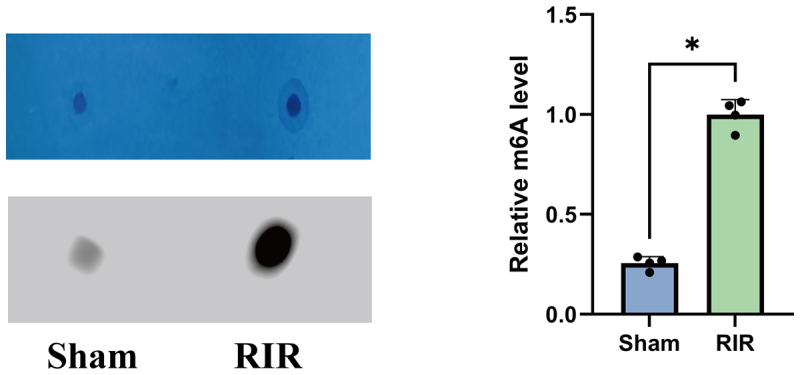
The RIR group exhibited markedly stronger m^6^A immunoreactive signals. The student’s t-test was used to assess the differences in m^6^A level between RIR group and Sham group (*n* = 4). Data are presented as mean ± SEM. *p < 0.05, **p < 0.01, ***p < 0.001, ****p < 0.0001; ns, not significant.

We used RT-qPCR to verify the transcriptome sequencing results by examining the expression of *Irx4*, *Kdr*, *Lyz2* from the intersecting mRNAs. The RT-qPCR results were consistent with the RNA-Seq data (Figure 8), confirming the significant differential expression (*p* < 0.05). An m^6^A dot blot assay was performed using total retinal RNA. Compared with the Sham group, the RIR group exhibited markedly stronger m^6^A immunoreactive signals (Figure 9).

## Discussion

In the mouse model of RIR, we observed a significant upregulation of core m^6^A regulatory enzymes together with widespread alterations in m^6^A methylation and mRNA expression profiles. These findings may indicate that m^6^A-associated post-transcriptional regulation is broadly involved in the RIR injury.

### Dysregulation of m^6^A regulators in RIR injury

To investigate the role of m^6^A modification in RIR injury, we first examined the expression of key m^6^A regulators over time. Our results showed that mRNAs encoding the methyltransferases (*Mettl3*, *Mettl5*) and the demethylases (*Alkbh5*, *Fto*) were all significantly upregulated on Day 1 and Day 3 following RIR injury, compared to the Sham group. On Day 7, however, the expression levels of those m^6^A regulators were no longer significantly different from those in the Sham group.

Previous studies converge on a common principle: m^6^A writers and erasers serve as post-transcriptional switches that calibrate inflammatory mRNA stability and downstream cell-death or senescence programs. For instance, In a model of cerebral I/R injury, METTL3 was significantly upregulated [17]. Functional inhibition of METTL3 protected neurons from the injury-induced mitochondrial dysfunction and ferroptosis. Mechanistically, METTL3 interacted with YTHDC1 to promote the m^6^A modification of SLC7A11, thereby reducing its stability. The subsequent knockdown of SLC7A11 mimicked the effects of METTL3 upregulation, confirming its involvement in mediating mitochondrial dysfunction and neuronal ferroptosis. Interestingly, previous studies have reported inconsistent expression patterns of Mettl3 in glaucoma animal models. One study showed that Mettl3 expression was downregulated in a high intraocular pressure‑induced retinal ischemia‑reperfusion (RIR) model, and this was associated with autophagy regulation [18]. In contrast, another study using an NMDA‑induced glaucoma model revealed that Mettl3 expression was upregulated, accompanied by enhanced ferroptosis [19]. These discrepancies among studies may be attributed to mild differences in the injury paradigms, the dominant cell death pathways, the cellular composition of the retina, and the temporal dynamics following injury. Previous studies have identified METTL5 as an oncogenic factor in clinical cancer samples, promoting cell proliferation, migration, and invasion [20,21]. However, the contribution of Mettl5 to epitranscriptomic regulation in eye-specific pathologies has not been systematically explored. Similarly, Alkbh5 has been shown to be involved in the inflammatory mechanisms of renal ischemia/reperfusion injury [12]. Alkbh5 regulates CCL28 mRNA stability by controlling its m^6^A modification. It regulates CCL28 mRNA stability through m^6^A modification, and its inhibition leads to increased CCL28 mRNA stability, which subsequently induces regulatory T cells (Tregs) and modulates the Tregs/inflammatory cell axis to improve renal function. FTO demethylase activity serves as a determinant of IL-1β mRNA stability and an activator of RPE senescence, and its suppression is sufficient to restrain both IL-1β abundance and the senescence [22].

Collectively, emerging evidence demonstrates that m^6^A writers and erasers function as stress-responsive modulators of inflammatory mRNA stability in above researches.

Consistent with this paradigm, the rapid induction of Mettl3, Alkbh5, and Fto following RIR injury suggests that these m^6^ A regulators may participate in the early epitranscriptomic response to retinal stress. Their dynamic expression changes support their potential roles as context-dependent candidate mediators of injury-associated RNA methylation remodeling. Future investigations may clarify whether modulation of these m^6^ A pathways can influence retinal injury progression and recovery.

### Genome-wide distribution of differential m^6^A modifications

Analysis of chromosomal distribution demonstrated that differential m^6^A peaks were widely distributed across the genome, with no obvious enrichment restricted to a particular chromosome. Although chromosomes 2, 7, and 11 exhibited relatively higher numbers of differential peaks, this pattern likely reflects differences in gene content rather than chromosome-specific targeting. These findings indicate that retinal ischemia – reperfusion injury is associated with broad epitranscriptomic remodeling affecting multiple genomic regions and biological processes.

### Integrated analysis of mRNA methylation and expression

We integrated MeRIP-seq and RNA-seq data to identify differentially methylated mRNA transcripts with corresponding differential expression. These findings indicate a potential positive relationship between RNA methylation levels and gene expression. It has been shown that m^6^A modification can affect stability of mRNA, and its effect on translation can also be stimulatory [23] or inhibitory [24,25]. The specific functional consequence is likely determined by the location of the methylation mark and the cellular circumstance. For example, during cell stress, the m^6^A-mRNAs degradation pathway is often suppressed, which stabilizes m^6^A-mRNAs and elevates the expression of stress-response transcripts [26]. This positive correlation between m^6^A methylation and mRNA expression was also observed in a rat balloon injury model of vascular injury [27]. Further research is needed to study these possibilities.

### Identification of key genes and functional pathways

We corroborated sequencing reliability through RT-qPCR validation of key m^6^A-dysregulated mRNAs, including *Irx4*, *Kdr*, and *Lyz2*, demonstrating consistent expression directionality with the integrated multi-omics results. Genes that showed significant alteration in both expression and m^6^A methylation are candidates of target genes.

A large proportion of intersecting mRNAs were both hypermethylated and upregulated (*n* = 598). Among these, *Lyz2* showed the highest fold change of expression, suggesting their significant involvement in the pathological response. Previous research showed that *Lyz2*^cre^-mediated depletion of autophagy-related gene in microglia aggravated the neuroinflammation and dopaminergic neuron losses in the substantia nigra and exacerbated the locomotor deficit in α-Syn-overexpressing mice [28].

In addition, a subset of hypomethylated mRNAs, including *Irx4*, showed significantly decreased expression. *Irx4* signals are predominantly localized to a neuronal subset within the retina, as revealed by RNA probe-based in situ hybridization using an *Irx4*-specific RNA probe [29]. Functional assays demonstrated that Irx4 not only defines expression domain, but also guides structural organization of retinal axons in the optic fiber layer. *Irx4* expression promotes orderly axon trajectory formation in the optic fiber layer, thereby supporting proper neuro-retinal connectivity and bundle organization [30]. In the context of RIR retinas, downregulation of *Irx4* more likely reflects impaired refinement of retinal axon guidance, which may compromise neuro-retinal connectivity and structural organization.

A small part of intersecting mRNAs was significantly hypomethylated and upregulated, including *Kdr*, a gene known to encode the vascular endothelial growth factor receptor (VEGFR) [31]. Under hypoxia, retinal endothelial cells showed significant upregulation of Kdr protein, indicating activation of VEGFR-associated signaling. The β-adrenergic receptor agonist returned hypoxia-induced Kdr upregulation toward baseline and restored retinal structural integrity after RIR injury, suggesting Kdr serves as a node that links hypoxia-driven VEGFR activation to retinal vascular degeneration and thickness loss in this model [32].

Subsequent KEGG pathway analysis revealed that these differentially hypermethylated mRNAs are mainly enriched in ECM-receptor interaction and tight junction. In previous study of pseudoexfoliation glaucoma [14], hypermethylated genes are mainly involved in ECM. The extracellular matrix, which comprises at least a third of tissue structures, has a mutually dependent relationship with immune system [33]. Shi et al. [34] found that vascular amyloid beta protein deposits were detected in retinas of mild cognitively impaired and Alzheimer’s disease (AD) patients, revealing that the retinal vascular tight junctions were compromised and linked to AD status.

This study has several limitations. First, the sample size was relatively limited. Second, MeRIP-seq and RNA-seq were performed only at Day 3 after RIR injury; therefore, the present data provide a single-time-point epitranscriptomic snapshot rather than a comprehensive characterization of the temporal dynamics of m^6^A methylation changes. Future multi-time-point epitranscriptomic profiling will be required to determine how m^6^A modifications evolve during injury progression and recovery. Third, in vivo loss-of-function validation of m^6^A methylation was not performed. Therefore, future studies should incorporate genetic or transcript-specific perturbation approaches, including m^6^A regulator loss-of-function models, m^6^A-site disruption strategies, and pathway-level interventions in RIR retinas, to establish causal relationships, define mechanistic hierarchies, and evaluate the therapeutic translational potential of m^6^A-mediated regulation.

## Data Availability

Data are available from figshare (DOI: 10.6084/m9.figshare.31881697). The raw sequencing data generated in this study have been deposited in the NCBI Gene Expression Omnibus (GEO) dataset under accession number GSE333759.
